# The Importance of Biologic Knowledge and Gene Expression Context for Genomic Data Interpretation

**DOI:** 10.3389/fgene.2018.00670

**Published:** 2018-12-18

**Authors:** Michael T. Zimmermann

**Affiliations:** ^1^Bioinformatics Research and Development Laboratory, Genomic Sciences and Precision Medicine Center, Medical College of Wisconsin, Milwaukee, WI, United States; ^2^Clinical and Translational Sciences Institute, Medical College of Wisconsin, Milwaukee, WI, United States

**Keywords:** precision medicine, genomic interpretation, variant prioritization, mechanistic modeling, knowledge generation

## Abstract

**Background:** Genomic sequencing, including whole exome sequencing (WES), is enabling a higher resolution for defining diseases, understand mechanisms, and improving the practice of clinical care. However, WES routinely identifies genomic variants with uncertain functional effects. Furthering uncertainty in WES data interpretation is that many genes can express multiple transcripts and their relative expression may differ by body tissue. In order to interpret WES data, we not only need to understand which transcript is most relevant, but what tissue is most relevant.

**Methods:** In this work, we quantify how frequently differences in transcript and tissue expression affect WES data interpretation at gene, pathway, disease, and biologic network levels. We combined and analyzed multiple large and publically available datasets to inform genomic data interpretation.

**Results:** Across well-established biologic pathways and genes with pathogenic disease variants, 54 and 40% have a different protein coding effect by transcript selection for, respectively, 25 and 50% of the genes contained. Additionally, strong differences in human tissue expression levels affect 33 and 19% of the same set of pathways and diseases for, respectively, 25 and 50% of the genes contained.

**Conclusion:** Whole exome sequencing identifies genomic variants, but to interpret the functional effects of those variants in high-resolution, we recommend building transcript selection and cross-tissue gene expression levels into hypotheses and analyses. Using current large-scale data, we show how extensively interpretation of genomic variants may differ according to transcript and tissue, across most pathways and disease. Thus, their inclusion is necessary for WES data interpretation.

## Introduction

Variety is a hallmark of BigData. In large-volume genomics such as whole exome sequencing (WES), we not only observe a variety of DNA variant types, but also may access a variety of data for variant annotation. “Annotation" refers to integration and mapping data to existing knowledge resources. Data integration is, for example, combining WES and gene expression data to study how genomic features may influence gene expression levels (Clyde, 2017) or how variants alter transcription factor binding sites (Mathelier et al., 2015). An example of using knowledge resources would be associating variants to known biochemical pathways. Annotation is necessary for biologists to understand how genetics influences physiology and for clinicians to understand how genomics data from individual patients may affect health and disease. Annotation and data integration are critical for prioritization and interpretation of WES data.

**FIGURE 1:**
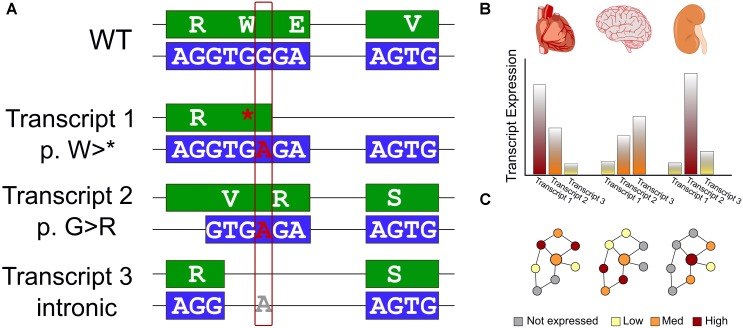
Interpreting the effects of genomics variants identified in WES requires transcript selection and context. **(A)** Schematic of a single genomic variant within a protein-coding gene that has a different impact in each of three isoforms. We first show a reference WT sequence with exons shown in boxes and introns as connecting lines. The DNA sequence is colored blue and encoded protein sequence in green. We consider the effect on this example sequence of a G > A variant. In the first transcript, the variant introduces an early stop codon. The second, an alternative splice site is used leading to a different reading frame, and a missense variant. Finally, for a third transcript an additional splicing pattern skips over the altered region and the genomic variant has no effect on the encoded protein sequence. **(B)** Many genes exhibit variability in expression level across tissues. This must also be considered for an accurate assessment of the effect of genomic variants. **(C)** Data interpretation is further challenged by the reality of biologic networks – the effects of transcript selection and tissue-specific expression affect not only the gene of interest (represented by a larger circle), but also the genes interacting with it (represented by smaller circles) in biologic networks or protein complexes. Thus, even if these features do not directly affect a gene of interest, a genomic variant may have a different apparent functional effect due to different environments in different biologic contexts.

One of the first annotations used in both prioritization and interpretation is what effect the variant has on a protein coding sequence. A variety of genomic variant types identified from WES, including single nucleotide variants (SNVs) and small insertions and deletions, can have drastic (e.g., frameshift), moderate (missense), or mild (silent) effects on the encoded protein. Even within missense SNVs, there is often a tremendous range of functional effects spanning from loss of stability, through impaired activity, to no measurable change to the protein. Our ability to predict functional changes to the protein depends on which transcript is used for annotation (Figure 1A). Additionally, gene regulation can supersede some of these effects – higher expression may compensate for a variant that lowers enzyme efficiency, while a gain-of-function variant may not be expressed (Figure 1B). As clinical genomics sequencing and direct-to-consumer testing become more prevalent, it is necessary to update current practices. One such current practice is to associate genomic variants to pathways, without accounting for biologic context – how the pathway may be different in terms of transcripts used and gene expression levels in different tissues and at different times. Understanding the functional effects of genomic variants requires the right context.

After variant annotation, researchers are often interested in a functional context; what biologic processes or functional pathways are affected? Genes do not act in isolation. The environment of a gene may differ between tissues or over time, and it may only be a few (or a single) of those contexts that the genomic variant has an effect (Figure 1C). In many WES studies, gene expression for the right tissue and in the right condition is typically not available, nor is a closely matched gene expression control. Therefore, it is crucial to bring in additional knowledge to assess which genes in these pathways may be most relevant.

In this work, we first gathered multiple publically available datasets to assess the question of how frequently known variants identified from WES would have a different interpretation due to transcript selection. Next, we quantified how frequently transcript selection and differential gene expression affect the genes within pathways and disease-gene networks. We also considered protein–protein interaction network features for affected versus unaffected genes. Our results emphasize how common both effects are and the need for improved methods to handle them. We believe that better addressing transcript selection and cross-tissue gene expression will increase the yield of WES data interpretation.

## Methods

### Reference Data

We downloaded the ClinVar database of genomic variants and their disease annotation (Landrum et al., 2014), May 2018 release for human genome build GRCh37. Variants were retained if they had at least one submitter that provided a manually curated assertion criteria. We further defined pathogenic variants as those with clinical significance categorized as (likely)pathogenic and lacking any other conflicting classification. We defined as VUS, variants with “uncertain” or “conflicting” annotation. We defined benign variants as those with a “benign” significance, or with “likely benign” as long as at least one submitter also classified it as “benign.” We included variants whose effect in ClinVar could reasonably change protein coding potential between different transcripts of the same gene, using a biotype filter including four categories: “protein coding,” “nonsense mediated decay,” “retained intron,” and “processed transcript.”

We downloaded pathway definitions from three resources: MSigDB Hallmarks (Subramanian et al., 2005) KEGG (Kanehisa et al., 2012), and Reactome (Croft et al., 2011). Pathways describe cellular processes with defined inputs and outputs. These three resources were chosen because they are publically available, commonly used, and represent, respectively, a small, medium, and large number of pathways and, respectively, broad, focused, and granular detail. We also downloaded three resources that define the relationships between genes and diseases: DisGeNet (Pinero et al., 2015), Monarch Initiative (Mungall et al., 2017), and Orphanet (INSERM, 1997). These three gene-centric definitions of diseases have been developed with different emphases. They are each more popular than others in different research areas, motivating us to consider how transcript-affected genes may distribute among them. We downloaded two recently developed resources of high-throughput and high-quality protein–protein interactions: CCSB (Rolland et al., 2014) and BioPlex (Huttlin et al., 2015). Protein physical interaction networks assess all potential interactions that each protein can make. They are more general than pathways and used to assess cross talk between pathways or broad patterns across the human proteome.

### Transcript Analysis

We used SnpEff (Cingolani et al., 2012) v4.3 and the Ensembl (Yates et al., 2016) database of transcript definitions to annotate the protein-coding effect of genomic variants. We annotated all transcripts meeting the above variant filtering criteria in order to be comprehensive (expression levels of these transcripts is considered below). We used chi-squared tests to compare the proportion of genes with differing impact across transcripts and for each variant type (pathogenic, VUS, and benign). We considered four classes of variant impact: high (alteration to coding length or frame), moderate (missense), low (silent), and modifier (non-coding). We define a gene as “transcript-affected” if the protein-coding impact of known pathogenic variants differs between the gene’s transcripts (Figure 1A). That is, we minimally required, for example, at least one transcript with a missense or nonsense variant, and a second transcript for the same variant with a different impact class.

### Tissue Enrichment Analysis

We used gene-level tissue enrichment from the human protein atlas (Uhlen et al., 2015, 2016). We used transcript-level data from the GTEx Consortium (2015) v7. We used ANOVA to assess intra- and inter-tissue gene expression variability across the 11,688 GTEx samples. To identify the largest effects, which we assumed to be the most robust, we define a gene as “expression-affected” if (using the most highly expressed transcript per gene) its expression was ≥80^th^ percentile of genes, the statistical significance for inter-tissue transcript expression differences was *p* < 1 × 10^-30^, and the inter-tissue ANOVA variance was ≥10x the intra-tissue variance (Figure 1B). More transcripts were statistically significant in the GTEx dataset than meet these criteria, even after multiple testing correction, but we focused on the genes expressed robustly and that have stark differences between tissues, assuming that these observations are the most likely to be reproducible and generalizable.

### Software Used

All analyses were performed in the R programming language (R Core Team, 2014). Pathway and network data were organized and queried using the Bioconductor package, RITAN (Zimmermann, 2018) v1.5.3. Graph metrics were computed using the igraph package (Csardi and Nepusz, 2006) v1.2.2. We generated plots using the R packages eulerr v5.0.0 and ggplot2 v3.1.0.

## Results

### Transcript Selection Alters Interpretation

In the Clinvar dataset, our inclusion criteria selected 46,804 pathogenic variants affecting 10,572 protein-coding transcripts (20,288 in total) from 3,439 genes. For VUS, we identified 156,782 variants affecting 14,847 protein-coding transcripts (29,174 in total) from 4,914 genes. Benign variants numbered 39,637 and affected 12,705 protein-coding transcripts (25,161 in total) in 4,324 genes. We tested if the proportion of transcript-altering variants was different for pathogenic, VUS, and benign variants. Pathogenic variants and VUS have a statistically significant higher proportion of transcript-affected genes (*p* < 1 × 10^-16^), but by a modest (∼0.05) effect size. We defined the 3,439 genes with pathogenic variants that have a different coding impact between transcripts, as transcript-affected.

**FIGURE 2:**
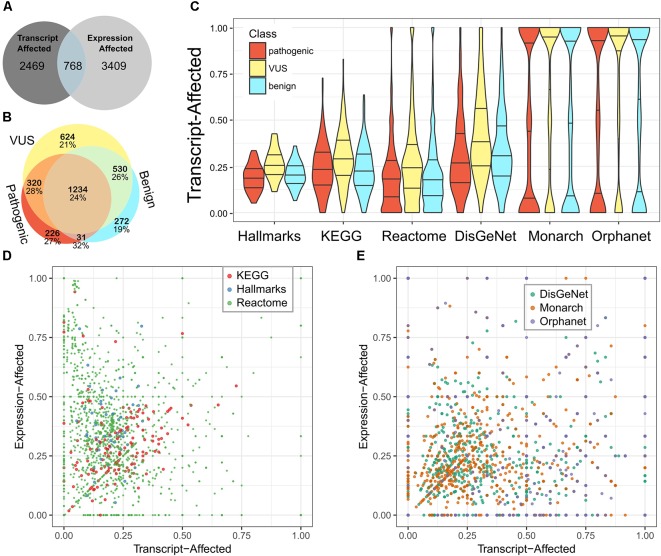
Most pathways and diseases are affected by variability in transcript selection and tissue-specific expression. We identified genes affected by each feature and calculated the fraction of genes affected within pathways or defining diseases. **(A)** We compare the number of genes defined as transcript-affected and expression-affected. **(B)** Among transcript-affected genes, we distinguish among the classification of variants. Most transcript-affected genes are affected by pathogenic or VUS. The percent of each genes that are also expression-affected is shown below the count, for each category of gene. **(C)** Across three resources that define biologic pathways (Hallmarks, KEGG, and Reactome) and three resources that define diseases (DisGeNet, Monarch, and Orphanet), the fraction of expression-affected genes varied significantly, but averaged to about 25%. We show the distribution of pathways’ fraction of genes affected using a violin plot (smoothed histogram) with quartiles indicated by horizontal lines; the middle line is the median. Genes affected by each of three classes of variants are distinguished. **(D)** For the three resources of biologic pathways, we show the more nuanced relationship between expression-affected and transcript-affected; each point in the plot is a pathway. Because there are many more Reactome pathways than the other two resources, points representing Reactome pathways are smaller so that they do not fully occlude others. **(E)** We similarly summarized diseases. There are multiple diseases for which every contributing gene is expression- or transcript-affected.

We used the fraction of transcript-affected genes within biologic pathways and definitions of genetic diseases as a simplified metric to assess (Figure 2A). While variability across pathways and diseases was high, the fraction of transcript-affected genes can be greater than 90%. On average, each disease has 68% of its genes transcript-affected and 29% of genes within pathways are similarly affected. The proportion of transcript-affected genes was higher for resources that were developed specifically for genetic and diagnostic audiences (e.g., Orphanet), compared to those developed for a broad audience (e.g., DisGeNet).

### Case Examples of Transcript-Affected Genes

To better understand the functional associations for transcript-associated variants, we selected three example proteins. First, CHD7 is a chromatin-remodeling enzyme whose dysfunction through genetic variants is well-established (Lalani et al., 2006). Previous studies have investigated two transcripts of CHD7 and demonstrated that each has a different biologic function (Colin et al., 2010; Kita et al., 2012). Therefore, how genetic variants may affect each of the two transcripts of CHD7 is critical to their interpretation. Both transcripts are highly expressed in the cerebellum and lowly expressed in multiple additional tissues. Dozens of pathogenic truncating and frameshift variants occur in the longer transcript that are non-coding in the shorter transcript. For example, Chr8:g.61693628C > T indicates p.Gln579^∗^ in the longer transcript and is intronic (c.1716+19C > T) in the shorter. Second, ARID1A is part of a chromatin-remodeling complex and has a multiple alternative transcripts that are expressed in multiple tissues. For the canonical transcript, the genomic variant Chr1:g.27099885G > A leads to a missense substitution, p.Gly1255Glu, while this exon is not used in some of the alternative transcripts. In this example, a missense variant could have little effect on a phenotype if the phenotype is primarily driven by the short transcript. Third, KMT2C, also known as MLL3, is a transcriptional regulator through histone methylation. KMT2C contains a structural domain called a PHD domain that binds methylated lysine residues on histone tails. Binding to histones is critical for regulating function. There are multiple transcripts of KMT2C. The pathogenic genomic variant, Chr7:g.151836877C > T, alters splicing in the canonical transcript. However, there are alternative and expressed transcripts that can be expressed to a higher level than the canonical transcript, for which this genomic variant precedes the coding region; the variant is within the 5′ untranslated region. Thus, determining precisely which transcript(s) is the right transcript for the right tissue at the right time is challenging, but necessary for improving genomics data interpretation, particularly when different transcripts may have different biologic functions.

### Effects of Genomic Variants Are Context Dependent

We used large publically available datasets to determine how frequently gene expression differences between human tissues significantly affects interpretation (expression-affected) and concordance with transcript-affected genes. Further, we investigated the neighbors of expression-affected genes in biologic pathways and networks. We defined 3,471 genes as expression-affected. While this number is similar to the number of genes defined as transcript-affected for pathogenic variants, the overlap is modest – 677 (20%) genes are in common. Because we chose conservative criteria for defining expression-affected genes, we do not expect to recapitulate all gene-level data from previous studies that aimed to characterize broad differences. Comparing to the Protein Atlas datasets, our conservative definition of expression-affected genes capture 27% of genes with moderate (grouped expression) to strong (enhanced/enriched expression) cross-tissue differences. Thus, the true impact of this feature is broader and we are focusing on the strongest signal.

Next, we looked up these genes in pathway resources and in resources that define the genetic contributors to diseases. There are some pathways and diseases that have no affected genes, but some for which every gene is affected (Figures 2B,C). On average, each disease and pathway has, respectively, 24 and 33% of their contributing genes expression-affected.

On average, pathways have a higher fraction of expression-associated genes than transcript-associated, while rare diseases have a closer balance between the two classes (Figures 2D,E). The diverse balance of transcript- and expression-affected genes means that each pathway and disease must be individually assessed.

We next considered network-based context for genes strongly affected cross-tissue gene expression. We measured network properties for expression-associated genes and compared to those from random sampling of the same number of protein-coding genes. We found a significant difference in degree distribution; In randomly generated graphs the number of genes connected to *x* other genes decayed at a rate of *x*^-*3.52*±*0.16*^, but in the expression-associated network the rate was *x*^-*2.72*^. The expression-associate network also has higher betweeness and edge density, compared to randomly generated graphs. Thus, consistent with prior data indicating that we are focusing on the strongest signal, the genes selected are representative from across large biologic interaction networks. They are likely to have an influence on function whether or not genes of interest are altered, because they will act in a different context, even within the majority of biologic pathways.

To summarize the prevalence of these two features across biologic networks and the genetic contributors to diseases, we calculated how many of them are affected for 25 or 50% of their associated genes. First, 54 and 40% have the interpretation of the protein coding impact changed by transcript selection for, respectively, 25 and 50% of the genes contained. Second, 33 and 19% of the same set of pathways and diseases are affected by the strong differences in human tissue expression levels for, respectively, 25 and 50% of the genes contained.

## Discussion

The complexity of interpreting the functional implications of genomic changes has been appreciated since before the completion of the Human Genome Project (Frazer, 2012). While data, methods, and tools have increased, our understanding of how deep these interpretation challenges are has also increased. While tissue-(2015) and transcript-specific (McCarthy et al., 2014) differences are expected in some biologic contexts, their prevalence across biologic pathways and their potential effects on WES data interpretation, are not systematically considered.

To interpret WES data, a variant’s impact is its effect on the coding potential of a transcript and must be distinguished from its functional effect and clinical actionability – all three are distinct. High-impact coding variants are likely to be loss-of-function. Moderate-impact coding variants (missense or in-frame INDELs) may have damaging effects on protein function or have tolerated effects. Even low-impact variants can be functional through alteration of regulatory motifs. A variant’s clinical significance is its functional significance that is relevant a human patient, in a particular clinical context (not necessarily the context in question). The question of whether a variant is “actionable” or not must be highly tailored to patients by their care team and within the context of their ongoing clinical care. These are three distinct layers of information.

Each patient may have other factors in their germline, development, lifestyle, or environment that either exacerbate or ameliorate a functional effect. Thus, we need finer context-specific resolution about how variants act together to impact cellular and physiologic processes. Transcript selection is a critical context to consider for variants of all types. Even for established benign variants, if a different transcript is relevant in a new study, the “benign” label may not be transferrable. In the context of a different transcript, its impact on the encoded protein may change. Additionally, transcript-affected variants may be expressed at a low level, further complicating their experimental assessment. In order to generate mechanistic understanding, we need robust methods for each of the three layers of effects.

In addition to robust analytic methods, health care providers need better tools to deliver salient genomics knowledge in timely and appropriate ways. Clinical genomics testing is becoming increasingly common for a variety of disease areas (Okur and Chung, 2017). An important extension of clinical genomics testing is to move beyond associations and to develop mechanisms. That is, many germline variants are associated with common diseases such as asthma or heart disease, but there is no clear functional link between the genotype and phenotype. Thus, the causal relationship between the genotype and phenotype is not established. In some diseases, the causal mechanism is clearer than for other diseases. For example, germline variants in certain DNA repair genes are associated with lifetime cancer risk because they increase the rate of variant accumulation across the genome, increasing the probability of inactivating a tumor suppressor or activating a proto-oncogene. Beyond direct genomic effects, many variants will have epigenetic effects with differences in cross-tissue expression profiles being one of the ways that epigenetic effects manifest. Previous studies have analyzed splice-QTLs in selected tissues or Li et al. (2016) across lymphoblastoid cell lines (Li et al., 2016; Takata et al., 2017). Thus, learning health systems need to be equipped to adapt to the new and increasingly varied data that is available to augment genomics data and aid its interpretation.

There are approaches for integrating existing data into more accurate knowledge models, but we need additional details to better interpret high-resolution data. Work by us (Zimmermann et al., 2017; Zimmermann et al., 2018) and others (Prokop et al., 2015; Towse et al., 2017; Agrahari et al., 2018) turns toward molecular modeling as the next frontier in genomics data interpretation, for its ability to not only indicate if a variant has an effect on the encoded molecule, but how and why. Methods for network-based integration (Dimitrakopoulos et al., 2018) and metabolic modeling (Nielsen, 2017) will enabling researchers to tune models to the data available for each sample. Additionally, the challenge remains for generating and linking the granular models to a systems- or physiologic-level model. Bringing data interpretation to a physiologic level will require a new high-resolution data type – high-resolution phenotyping.(Müller et al., 2018) The many types of additional data we have discussed all enhance the interpretability of data generated by exome sequencing and will likely lead to greater clinical applicability of genomics data.

## Conclusion

We have quantified the prevalence across biologic pathways and disease definitions, of changes in the interpretation of genomic variants due to different protein coding impact across transcripts, and of the encoded genes’ expression differing between human tissues. Not only is WES data part of the BigData in genomic medicine, but the volume and variety of annotation resources makes them critical components too. Clinical genetics sequencing is increasing as part of Precision Medicine, increasing the demand for methods that interpret WES data from individual patients. Leveraging multiple large and publically available datasets, our analysis highlights the variety of data and methods needed, to interpret WES data for new biologic or disease-specific use.

## Author Contributions

MZ designed the study, ran analyses, generated figures, and wrote the paper.

## Conflict of Interest Statement

The author declares that the research was conducted in the absence of any commercial or financial relationships that could be construed as a potential conflict of interest.
